# A hepatic abscess complicated with spinal infection and a psoas abscess: a case report and literature review

**DOI:** 10.3389/fmed.2026.1928987

**Published:** 2026-10-21

**Authors:** Xingsheng Yu, Hao Xing, Zhengqi Chang

**Affiliations:** Department of Orthopaedics, The 960th Hospital of PLA, Jinan, China

**Keywords:** case report, diabetes, hepatic abscess, spinal infection, vacuum sealing drainage

## Abstract

**Background:**

Dissemination of bacteria from a *Klebsiella pneumoniae* liver abscess through the bloodstream can occur, with the rare clinical occurrence of concurrent multilevel spinal epidural infection and a psoas abscess, which pose a high risk of disability. This report describes a case of complicated spinal infection secondary to a liver abscess.

**Case presentation:**

A 65-year-old male patient with type 2 diabetes and a history of *K. pneumoniae* liver abscess subsequently developed multilevel epidural infection, discitis, osteomyelitis, and psoas abscess, with the pathogen being a subspecies of *K. pneumoniae*. The patient underwent multiple staged surgical debridements and received combined repeated vacuum sealing drainage (negative-pressure wound therapy). In the neurological function assessments, the patient was admitted as American Spinal Injury Association (ASIA) C, the lowest grade, and discharged as ASIA E. At the latest follow-up, the patient had good infection control, could walk short distances with a thoracolumbosacral orthosis, and had no recurrence of infection.

**Conclusion:**

In diabetic patients with a *K. pneumoniae* liver abscess, clinicians should be vigilant for hematogenous spinal infections. For critically ill patients with extensive multilevel spinal infections, staged debridement combined with negative-pressure wound therapy is a feasible treatment strategy.

## Introduction

Spinal infection is a microbial infectious disease involving the vertebral body, intervertebral discs, and/or adjacent paravertebral soft tissues. The main causes include hematogenous spread, contiguous infection spread, and direct infection. Studies have shown that pyogenic spinal infections from hematogenous spread mainly affect the lumbar spine (58%), followed by the thoracic spine (30%) and cervical spine (11%) (1–3). Patients with diabetes and a hepatic abscess are at risk of septicemia or sepsis due to immunodeficiency caused by high blood sugar and liver dysfunction, which can lead to distant infections (4). This case involves a diabetic patient with a history of *Klebsiella* hepatic abscess presenting with 2 months of back pain exacerbated by weakness during walking for the previous 5 days. Magnetic resonance imaging (MRI) revealed infection signals in the spinal canal from thoracic vertebrae 5 to 11 and lumbar vertebrae 2 to 5, along with the left paravertebral area. A confirmed diagnosis of *K. pneumoniae* subsp. infection was made through biopsy, consistent with previous microbial findings in the hepatic abscess. This suggests that bacteria from the hepatic abscess may have spread via the bloodstream, causing infection in the corresponding spinal segments.

## Case presentation

A 65-year-old male patient with a 2-year history of diabetes was diagnosed with a liver infection caused by *K. pneumoniae subsp. pneumoniae*. He had undergone a liver abscess puncture biopsy at a local hospital 2 months previously. Presenting with worsening lower back pain over the past 2 months and weakness in both legs for 5 days while walking, he sought medical attention. Upon examination, he was found to have limited flexion and extension of the lumbar spine, with tenderness and percussion pain in the spinous processes and interspinous spaces of the thoracic and lower lumbar vertebrae. The strength of the bilateral tibialis anterior and hamstring muscles was graded as 1/5, and the quadriceps strength was graded as 0/5. The American Spinal Injury Association (ASIA) classification was C. MRI results revealed infection signals within the spinal canals of thoracic vertebrae 5–11 and lumbar vertebrae 2–5, as well as near the left vertebral body. Laboratory findings upon admission showed a white blood cell count of 8.63 × 10^9^/L, neutrophil count of 8.10 × 10^9^/L, erythrocyte sedimentation rate (ESR) of 78 mm/h, and C-reactive protein (CRP) of 300 mg/L. Blood glucose-related indicators included a glycated hemoglobin level of 8.50% and a fasting blood glucose level of 10.31 mmol/L. The insulin treatment plan consisted of 8 units of insulin glulisine three times a day and 12 units of insulin glargine in the evening.

After completing relevant tests and examinations, the patient received treatment for infection with imipenem-cilastatin sodium and intravenous human albumin. Under ultrasound guidance, abscess drainage was performed on the sternum and spine percutaneously. During the procedure, 15 mL of pus was aspirated from the spinal muscles, and an 8F drainage catheter was inserted. Bacterial culture revealed *K. pneumoniae subsp. pneumoniae*. The next day, the infection worsened, with laboratory results showing an erythrocyte sedimentation rate of 87 mm/h, C-reactive protein of 248.60 mg/L, red blood cell count of 3.60 × 10^12^/L, hemoglobin of 98 g/L, platelet count of 62 × 10^9^/L, and albumin of 22.0 g/L. Due to severe thrombocytopenia and hypoalbuminemia, the patient was transferred to the intensive care unit for resuscitation. Treatment included imipenem for infection, ursodeoxycholic acid for inflammation, albumin and platelet transfusions, and anticoagulation. After stabilization, the patient was transferred back for continued treatment of the spinal infection. Due to deep vein thrombosis in the lower limbs, venous Doppler ultrasound and filter insertion were performed. Considering the patient's poor general condition, a vacuum sealing drainage (VSD)-based abscess drainage surgery was chosen. A VSD negative-pressure sponge was placed in the L5/S1 interspace to fill the infected cavity and assist with drainage. Subsequent surgeries involved removing the vertebral plates at T6/7 and L1/2 to eliminate the abscess and relieve dural compression. The final surgery under general anesthesia addressed the residual pus in the right paraspinal muscles. Following multiple surgical interventions and VSD therapy, the patient was eventually discharged after successful recovery. Follow-up at 2 years postoperatively showed the patient had regained self-care abilities, and local hospital reevaluation indicated normalization of the inflammatory markers CRP and ESR. The patient’s ASIA rating was E. For detailed VSD outcome measures, please consult our team's previous study (5) (Figure 1).

**Figure 1:**
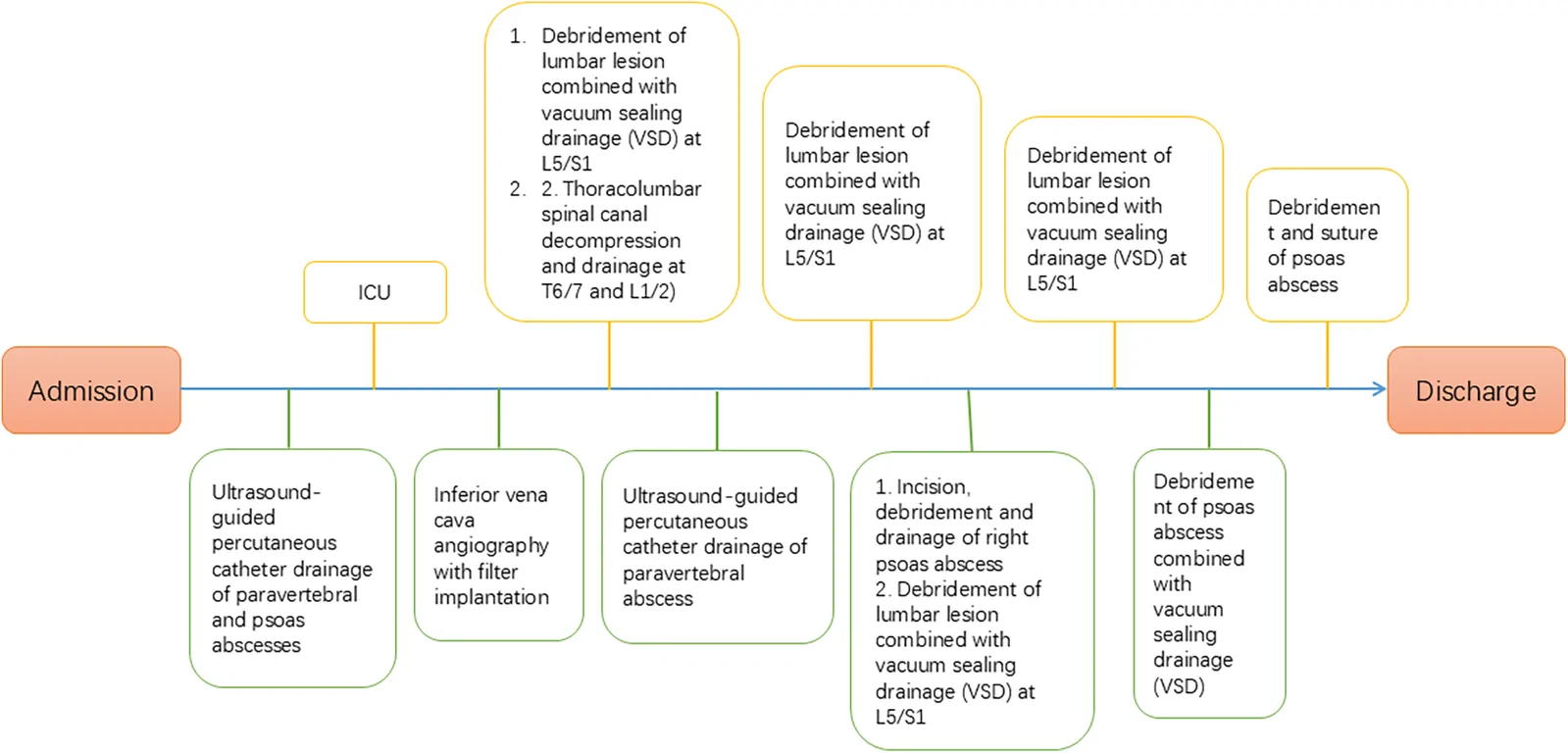
Major therapeutic interventions from admission to discharge.

## Discussion

Diabetic patients, due to their prolonged exposure to high blood sugar and suppressed immune systems, are highly susceptible to infections. Studies have shown that diabetic patients are 1.5–4 times more likely to develop infections compared to healthy individuals (6). Common organ infections include *Klebsiella* hepatic abscesses and cholecystitis (5, 7). If infections in diabetic patients are not effectively controlled, they can progress to systemic dissemination, sepsis, and even death (8, 9). Tsai et al. revealed that diabetic patients in Taiwan have a ninefold increased risk of developing pyogenic hepatic abscesses compared to non-diabetic individuals (10). Hepatic abscesses, as a source of systemic infection, can easily lead to infections in other organs or parts of the body. Kuramochi et al. (11) reported a case of a 56-year-old Japanese male patient who was hospitalized with a 10-day history of high-grade fever and upper abdominal pain. An ultrasound revealed a pyogenic hepatic abscess in the right lobe, which was drained, and cultures showed multidrug-resistant *K. pneumoniae*. On the 6th day of hospitalization, the patient developed paraplegia and urinary retention, with absent deep tendon reflexes in the lower limbs. MRI scans revealed epidural and paraspinal abscesses in the thoracic spine, necessitating emergency decompressive laminectomy, with cultures confirming multidrug-resistant *K. pneumoniae*. Duran et al. (12) reported a case of a 62-year-old male diabetic patient admitted with meningeal syndrome, with blood and cerebrospinal fluid cultures isolating *K. pneumoniae*. A CT scan revealed a hepatic abscess, suggesting bacterial dissemination to the cerebrospinal fluid. Soo et al. (13) described a case of a 34-year-old male patient admitted with a history of diarrhea, vomiting, fever, and cough. Abdominal ultrasound showed liver and spleen abscesses, with drainage cultures identifying *Burkholderia pseudomallei*. A week later, the patient developed back pain, and MRI confirmed an intrasacral abscess on the right side. The patient received 6 weeks of intravenous antibiotics plus oral co-trimoxazole therapy.

Chen and Li (14) reported a case of invasive *K. pneumoniae* liver abscess syndrome secondary to the L3 and L4 vertebral bodies and paraspinal soft tissue infection. Genomic sequencing confirmed the *K. pneumoniae* infection. Hsieh et al. (15) reported a 44-year-old male patient who presented with fever, chills, and diarrhea. Imaging revealed a 4.7 cm × 2.8 cm liver abscess with septations in the right lobe. Spinal MRI showed abnormal signal enhancement above C5 and C6 and at C7, with degenerative changes and epidural and prevertebral abscesses. Tissue culture identified *K. pneumoniae* and the patient was treated postoperatively with intravenous antibiotics. In our case report, the patient was admitted for back pain and weakness on walking and was diagnosed with secondary spinal infection, with a history of diabetes and liver abscess. The liver abscess was caused by a *K. pneumoniae* subspecies, with the spinal infection and psoas abscess also attributed to the same pathogen.

Spinal infections account for 2%–7% of all musculoskeletal infections (1, 3). The etiology includes hematogenous spread, contiguous spread, and direct inoculation. In our case, based on the patient's disease progression, the types of causative microorganisms, and the history of diabetes, we believe the spinal lesion was likely due to hematogenous spread from a *K. pneumoniae* hepatic abscess to the spine. Secondary spinal infections from infections in other parts of the body are not uncommon. For instance, Meneses et al. (16) reported a case of an 82-year-old male patient who developed a psoas abscess with *Staphylococcus schleiferi* 1 year after receiving an implantable cardioverter defibrillator (ICD) for heart disease. The infection progressed to *S. schleiferi* osteomyelitis 2 years post-surgery at the L3 and L4 vertebrae, with the ICD surface culture confirming the presence of the pathogen. In addition, urinary tract infections can also lead to secondary spinal infections. AlSabea et al. (17) reported a rare case of an epidural abscess due to *Staphylococcus aureus* following a urinary tract infection. Patients with secondary spinal infections often have underlying chronic conditions that compromise their nutritional status and immune function, making them susceptible to bacterial dissemination to the spine during distant body infections or surgical stress (Table 1).

**Table 1:** Published case reports of secondary spinal infections.

First author	Sex	Age	Primary disease	Site of spinal infection	Microorganism	Treatment	Comorbidities
Kuramochi (11)	Male	56	Liver abscess	Vertebral osteomyelitis (T8 and T9) and an epidural abscess	*K. pneumoniae*	Biapenem, levofloxacin, and posterior laminectomy decompression	Hypertension
Duran (12)	Male	62	Liver abscess	Meningeal syndrome and vertebral osteomyelitis (L4–S1)	*K. pneumoniae*	Ceftriaxone	Type 2 diabetes mellitus
Soo (13)	Male	34	Liver abscess and a splenic abscess	Vertebral osteomyelitis (S1)	*B. pseudomallei*	Meropenem, ceftazidime, and sulfamethoxazole-trimethoprim	Type 2 diabetes mellitus
Li (14)	Male	52	Liver abscess	Vertebral osteomyelitis (L3 and L4)	*K. pneumoniae*	Vancomycin, piperacillin-tazobactam, ceftazidime-avibactam sodium, and levofloxacin	Type 2 diabetes mellitus
Hsieh (15)	Male	44	Liver abscess	C5, C6, and C7	*K. pneumoniae*	Surgical debridement and ceftriaxone	N/A
AlSabea (17)	Male	54	Urinary tract infection	Vertebral osteomyelitis (C5–C6) and an epidural abscess (C6–C7)	*S. aureus*	(1) C6 corpectomy (more than 70%), evacuation of epidural abscess, and decompression of spinal cord and nerve roots; (2) cefazolin	Type 2 diabetes mellitus
Tran (18)	Male	66	Periodontal disease	Lumbar discitis and vertebral osteomyelitis (L4–5)	*Streptococcus cristatus*	Amoxicillin	Myasthenia gravis, hypertension, chronic kidney disease, and type 2 diabetes mellitus
Hashimoto (19)	Male	60	Infective endocarditis	Vertebral osteomyelitis (L4 and L5) and an epidural abscess	*Streptococcus sanguinis*	Penicillin and gentamicin	Hypertension and arrhythmia
Kadota (20)	Female	63	Pleurisy	Vertebral osteomyelitis (T7 and T8)	*Mycobacterium abscessus* ssp	Imipenem/cilastatin, amikacin, and clarithromycin	N/A
Luo (21)	Male	54	Subacute infective endocarditis	An intramedullary abscess and multiple brain abscesses	*Streptococcus anginosus*	Ceftriaxone, linezolid, and cefixime	N/A
Vasconcellos (22)	Male	62	Native valve endocarditis	Lumbar discitis (L1/2)	*Staphylococcus hominis*	Vancomycin	Type 2 diabetes mellitus, coronary atherosclerosis, and hypertension
Moustafa (23)	Male	69	Urinary tract infection	Vertebral osteomyelitis (C6 and C7) and an epidural abscess	*Escherichia coli*	Vancomycin, cefepime, acyclovir, and ceftriaxone; anterior cervical epidural abscess drainage, decompression, and fusion	Prostatitis

MRI in this case showed an epidural abscess in the spinal canal from thoracic vertebrae 5 to 11, severe destruction at the L5/S1 level, and a formation of an abscess in the left lumbar muscle area. The patient had a history of hepatic abscess treated with drainage before admission, with significant improvement seen on abdominal CT (Figure 2). Specific treatment for the spinal infection had not been initiated. Given the patient's history of hepatic abscess and antibiotic treatment, intravenous imipenem-cilastatin was started upon admission to control the infection. For the lumbar muscle abscess, ultrasound-guided percutaneous drainage was performed twice, along with surgical debridement and VSD therapy. The infection at the L5/S1 level was managed with posterior lumbar debridement and VSD placement, with two VSD replacements during the procedure. The epidural abscesses in the thoracic and upper lumbar spine were treated with decompression and drainage by vertebral plate removal at the L1/2 and T6/7 levels (Figure 3). Following the timely administration of sensitive antibiotics based on past experiences with *K. pneumoniae* hepatic abscesses, in conjunction with multiple surgical interventions, the patient was eventually discharged after a successful recovery.

**Figure 2:**
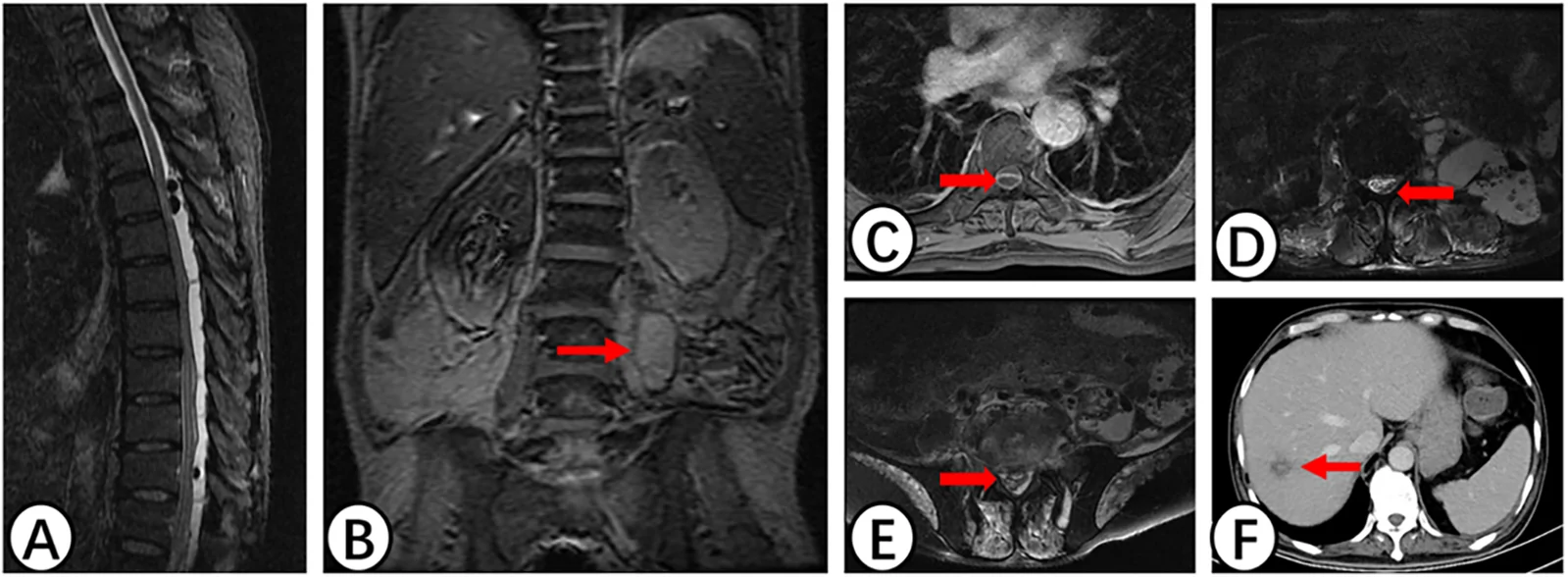
Imaging examinations on admission. **(A)** Sagittal thoracic MRI shows continuous long-segment epidural abscess signal within the thoracic spinal canal. **(B)** Coronal lumbar MRI reveals a left psoas abscess, indicated by the red arrow. **(C–E)** Axial MRI scans at the thoracic vertebra, psoas muscle, and L5/S1 disc levels, respectively, demonstrate abscess involvement at all levels with severe destruction of the L5/S1 intervertebral space. **(F)** Upper abdominal CT scan shows the primary hepatic abscess lesion, marked by the red arrow. All MRI scans were T2.

**Figure 3:**
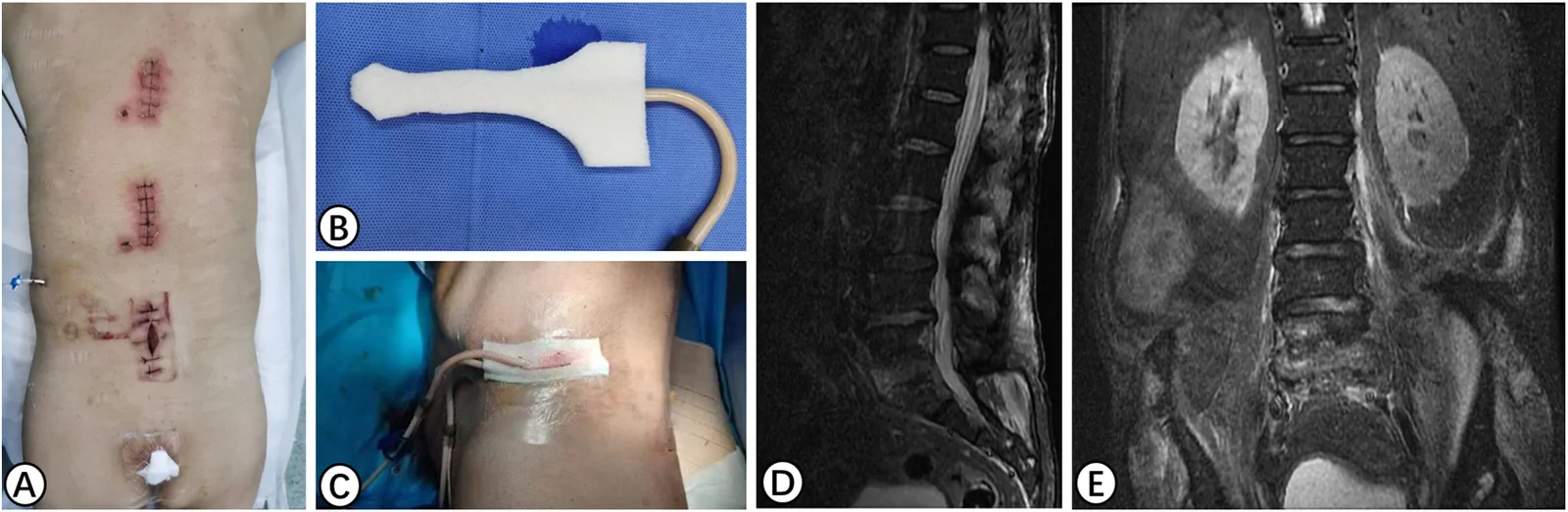
Intraoperative and postoperative imaging data. **(A)**. A drainage tube was inserted for ultrasound-guided percutaneous psoas abscess drainage in the left lumbar region. Furthermore, there were surgical decompression and drainage sites at T6/7, L1/2, and L5/S1, with VSD at L5/S1. **(B)** Trimmed VSD negative-pressure foam. **(C)** Open debridement of the psoas muscle with VSD placement. **(D,E)** MRI scans at discharge show remarkable improvement of the lesions at the L5/S1 intervertebral space and left psoas abscess compared with admission (more than 50 days had passed). All MRIs were T2.

Patients in this category typically present with predominant lower back pain in the early stages, which lacks specificity. The spinal infection site often only becomes apparent several weeks after the onset of the liver abscess. Clinicians tend to focus more on the primary liver infection site, potentially overlooking subsequent distant hematogenous spread to the spine. In addition, initial X-ray findings may show subtle bone destruction, further delaying diagnosis. When diabetic patients with a liver abscess experience new-onset lower back pain, clinicians should be vigilant and promptly arrange for spinal MRI. A review of relevant literature revealed the common characteristics of spinal infections secondary to abdominal or visceral infections. Regarding pathogens, although *S. aureus* is the most common pathogen in hematogenous spinal infections, gram-negative bacteria predominate in cases originating from abdominal infections, with *K. pneumoniae* being particularly prominent. Çolak et al. previously reported the world's first case of *Staphylococcus xylosus*-induced pyogenic sacroiliac joint arthritis with an abscess. The patient had only a mild increase in white blood cells (13.47 × 10^3^/μL), with normal inflammatory markers, including CRP and ESR. The treatment involved conservative management with needle drainage and long-term antibiotics (24). The lumbar spine is the most commonly affected segment due to its rich blood supply, followed by the thoracolumbar and thoracic spine. Multi-segment contiguous infections are not uncommon, often indicating a more severe condition and complex treatment. While antibiotics form the basis of treatment, a significant number of patients require surgical intervention, especially when complications such as an epidural abscess causing nerve compression or progressive spinal deformity occur. In cases where antibiotics alone may not adequately penetrate the abscess wall, such as in the extensive psoas abscess illustrated in our case, surgical debridement and drainage are necessary. The patient's ultimate prognosis is influenced by various factors, including control of underlying conditions (such as diabetes), timely diagnosis, and complete eradication of the infection. In terms of treatment, antibiotics are usually used empirically, and once the pathogen is identified, the treatment regimen is adjusted accordingly. Decompression surgery is generally reserved for patients with symptomatic nerve compression, severe sepsis, spinal instability, or unknown microbiological etiology, with specific surgical indications often influenced by local medical institution guidelines (3). The treatment strategy in this case significantly differs from previous reports, demonstrating innovation in the treatment approach for critically ill patients with spinal infections. Traditionally, severe spinal infections requiring surgery were treated with one-stage open lesion debridement, bone fusion, and internal fixation. However, for critically ill patients, such as the patient in this case who also had severe sepsis, thrombocytopenia, and hypoalbuminemia, the risks associated with a one-stage major surgery are extremely high. Extensive debridement and internal fixation can lead to significant bleeding, which patients with impaired coagulation function may not tolerate. In addition, in the presence of uncontrolled systemic infection, implanted internal fixation devices can easily become new sources of infection, leading to treatment failure.

This study combined previous experience in treating complex spinal infections and utilized a treatment approach centered around staged negative-pressure wound therapy. The core of this approach is “infection control first, followed by bony reconstruction.” Initially, thorough debridement was performed using minimally invasive or limited open surgery, followed by the placement of a negative-pressure drainage system. Negative-pressure drainage effectively and continuously removes pus and necrotic tissue, reducing bacterial load, improving local microcirculation, promoting granulation tissue growth, and creating favorable conditions for subsequent treatment. By prioritizing infection control through one or multiple debridement and negative-pressure device changes, the immediate high-risk internal fixation was avoided. During the infection control phase, we utilize rigid thoracolumbosacral orthoses to provide external support and maintain relative spinal stability in patients. Delayed phase two reconstructive surgery is reserved as an alternative approach for managing patients who develop progressive spinal instability or deformity post infection control. This staged treatment strategy is particularly suitable for critically ill patients with poor overall health who are unable to tolerate a large-scale reconstructive surgery in the first phase.

This study has some limitations. First, as it is a single-case report, its conclusions have limited generalizability. Second, although the patient in this case maintained spinal stability through orthotic external fixation, avoiding a second-stage fusion surgery, the long-term occurrence of spinal kyphosis or chronic pain still requires extended follow-up observation. Finally, the effectiveness and safety of this staged negative-pressure therapy strategy need to be compared to traditional single-stage surgery through larger prospective randomized controlled studies to draw more definitive conclusions.

## Conclusion

For diabetic patients with a history of lower back pain, especially those with a history of hepatic abscess, the possibility of secondary spinal infection should be considered and MRI should be conducted. Based on the sequence of disease progression, type of pathogenic bacteria, and the patient's condition, the type of spinal infection should be comprehensively assessed to provide timely and effective antibiotic therapy and surgical debridement. Negative-pressure wound therapy, as a technique for managing complex wounds, when used in combination, can significantly control the progression of the infection and reduce surgical stress. Patients with secondary spinal infections are often also diagnosed with chronic diseases such as diabetes and immune deficiency. Once diagnosed, prompt and aggressive treatment should be administered.

## Data Availability

The original contributions presented in the study are included in the article/Supplementary Material; further inquiries can be directed to the corresponding author.

## References

[1] Lener S Hartmann S Barbagallo GMV Certo F Thome C Tschugg A. Management of spinal infection: a review of the literature. Acta Neurochir (Wien). (2018) 160:487–96. 10.1007/s00701-018-3467-2 29356895 PMC5807463

[2] Bathini A Maxwell CR Hedayat H Barrett J Hakma Z. Case report: vertebral osteomyelitis secondary to a dental abscess. BMC Infect Dis. (2020) 20:133. 10.1186/s12879-020-4857-7 32050917 PMC7017548

[3] Phan VT Dimou J Davidson AS Giulieri S Silver J Yoon SW. Spinal infection: an evolving clinical and public health challenge. J Clin Neurosci. (2026) 147:111949. 10.1016/j.jocn.2026.111949 41740215

[4] Frydrych LM Bian G O'Lone DE Ward PA Delano MJ. Obesity and type 2 diabetes mellitus drive immune dysfunction, infection development, and sepsis mortality. J Leukoc Biol. (2018) 104:525–34. 10.1002/JLB.5VMR0118-021RR 30066958

[5] Xing W Yang Y Bai Y Yu X Chang Z. A comparison of negative pressure and conventional therapy in spine infections: a single-center retrospective study. J Pers Med. (2023) 13:162. 10.3390/jpm13020162 36836397 PMC9965435

[6] Holt RIG Cockram CS Ma RCW Luk AOY. Diabetes and infection: review of the epidemiology, mechanisms and principles of treatment. Diabetologia. (2024) 67:1168–80. 10.1007/s00125-024-06102-x 38374451 PMC11153295

[7] Wang JL Hsu CR Wu CY Lin HH. Diabetes and obesity and risk of pyogenic liver abscess. Sci Rep. (2023) 13:7922. 10.1038/s41598-023-34889-z 37193729 PMC10188555

[8] Cui J Liu Y Li J. The new changes of epidemiology, etiology, and clinical characteristics of pyogenic liver abscesses: a retrospective study in a hospital in northern China. Infect Drug Resist. (2023) 16:4013–23. 10.2147/IDR.S416860 37377730 PMC10292621

[9] Liu S Ge X Xu X Zhang K Lu X. Alterations of gut microbiota in pyogenic liver abscess patients with and without type 2 diabetes mellitus. Infect Drug Resist. (2024) 17:2149–58. 10.2147/IDR.S456423 38828371 PMC11144416

[10] Tsai FC Huang YT Chang LY Wang JT. Pyogenic liver abscess as endemic disease, Taiwan. Emerg Infect Dis. (2008) 14:1592–600. 10.3201/eid1410.071254 18826824 PMC2609891

[11] Kuramochi G Takei SI Sato M Isokawa O Takemae T Takahashi A. *Klebsiella pneumoniae* liver abscess associated with septic spinal epidural abscess. Hepatol Res. (2005) 31:48–52. 10.1016/j.hepres.2004.09.006 15652471

[12] Duran MV Landaburu MF Scarabino M Furst L J M. Meningitis and myelitis by hypervirulent *Klebsiella pneumoniae*. Medicina (B Aires). (2024) 84:975–8. 39399938

[13] Soo KC Lee KS Ooi SY Darwina A Sannasey S Lee HG. Disseminated melioidosis with spinal intraosseous abscess. Med J Malaysia. (2021) 76:251–3. 33742639

[14] Li F Chen X. Pulmonary abscess, endophthalmitis, and lumbar spine infection attributable to invasive *Klebsiella pneumoniae* liver abscess syndrome: a case report. Infect Drug Resist. (2026) 19:1–7. 10.2147/IDR.S569282 PMC13039657 41928899

[15] Hsieh MJ Lu TC Ma MH Wang HP Chen SC. Unrecognized cervical spinal epidural abscess associated with metastatic *Klebsiella pneumoniae* bacteremia and liver abscess in nondiabetic patients. Diagn Microbiol Infect Dis. (2009) 65:65–8. 10.1016/j.diagmicrobio.2009.05.013 19679238

[16] Meneses D Diaz-Pollan B G Pereda A A Fernandez F. Vertebral osteomyelitis secondary to a *S. schleiferi* infection from a cardiac defibrillator. Enferm Infecc Microbiol Clin. (2015) 33:133–4. 10.1016/j.eimc.2014.05.006 25617017

[17] AlSabea N Kanor U Garcia AS Shah A Sun A. Spinal epidural abscess of uncommon presentation following urinary tract infection: a case report. World J Clin Cases. (2025) 13:109027. 10.12998/wjcc.v13.i29.109027 40933934 PMC12417925

[18] Tran H Ai A Gallardo-Huizar OE Kahn M Mathisen G. Vertebral osteomyelitis secondary to *Streptococcus cristatus* infection. Heliyon. (2023) 9:e19616. 10.1016/j.heliyon.2023.e19616 37809561 PMC10558856

[19] Hashimoto M Ueda K Nakao T Tanaka T Komuro I. Vertebral osteomyelitis secondary to infective endocarditis detected by repeated magnetic resonance imaging: a case report. Eur Heart J Case Rep. (2023) 7:ytad552. 10.1093/ehjcr/ytad552 38426047 PMC10903173

[20] Kadota N Shinohara T Hino H Goda Y Murase Y Mitarai S. *Mycobacterium abscessus* ssp. abscessus infection progressing to empyema from vertebral osteomyelitis in an immunocompetent patient without pulmonary disease: a case report. BMC Pulm Med. (2019) 19:100. 10.1186/s12890-019-0860-4 31126264 PMC6534929

[21] Luo W Yin Y Liu W Ren H. Intramedullary spinal cord abscess with brain abscess due to subacute infective endocarditis. BMC Neurol. (2023) 23:18. 10.1186/s12883-023-03050-8 36647036 PMC9841633

[22] Vasconcellos D Weng B Wu P Thompson G Sutjita M. *Staphylococcus hominis* infective endocarditis presenting with embolic splenic and renal infarcts and spinal discitis. Case Rep Infect Dis. (2022) 2022:1–5. 10.1155/2022/7183049 PMC9124120 35607353

[23] Moustafa A Kheireldine R Khan Z Alim H Khan MS Alsamman MA. Cervical spinal osteomyelitis with epidural abscess following an *Escherichia coli* urinary tract infection in an immunocompetent host. Case Rep Infect Dis. (2019) 2019:1–5. 10.1155/2019/5286726 PMC6501268 31143482

[24] Çolak AF Yazar B Bucağa TM Demirel K Aksakal MF Yalçınkaya B. A rare case presentation of septic sacroiliitis caused by *Staphylococcus xylosus* and complicated with abscess formation: a case report. Diagn Microbiol Infect Dis. (2024) 109(3):116290. 10.1016/j.diagmicrobio.2024.116290 38643676

